# Inducible TDG knockout models to study epigenetic regulation

**DOI:** 10.12688/f1000research.25637.2

**Published:** 2020-10-19

**Authors:** Simon D. Schwarz, Eliane Grundbacher, Alexandra M. Hrovat, Jianming Xu, Anna Kuśnierczyk, Cathrine B. Vågbø, Primo Schär, David Schuermann

**Affiliations:** 1 Department of Biomedicine, University of Basel, Basel, 4058, Switzerland; 2 Proteomics and Modomics Experimental Core Facility (PROMEC), Norwegian University of Science and Technology, Trondheim, 7491, Norway

**Keywords:** Embryonic Stem Cells, TDG, Active DNA Demethylation, Base Excision Repair, Neuronal Differentiation, Minigene, Cre/loxP, Tamoxifen

## Abstract

Mechanistic and functional studies by gene disruption or editing approaches often suffer from confounding effects like compensatory cellular adaptations generated by clonal selection. These issues become particularly relevant when studying factors directly involved in genetic or epigenetic maintenance. To provide a genetic tool for functional and mechanistic investigation of DNA-repair mediated active DNA demethylation, we generated experimental models in mice and murine embryonic stem cells (ESCs) based on a minigene of the thymine-DNA glycosylase (TDG). The *loxP*-flanked *miniTdg* is rapidly and reliably excised in mice and ESCs by tamoxifen-induced Cre activation, depleting TDG to undetectable levels within 24 hours. We describe the functionality of the engineered *miniTdg* in mouse and ESCs (TDGiKO ESCs) and validate the pluripotency and differentiation potential of TDGiKO ESCs as well as the phenotype of induced TDG depletion. The controlled and rapid depletion of TDG allows for a precise manipulation at any point in time of multistep experimental procedures as presented here for neuronal differentiation *in vitro*. Thus, we provide a tested and well-controlled genetic tool for the functional and mechanistic investigation of TDG in active DNA (de)methylation and/or DNA repair with minimal interference from adaptive effects and clonal selection.

## Introduction

To allow for the differentiation to a multitude of cell types during the development of multicellular organisms, stem cells need to respond to a variety of extrinsic and intrinsic developmental cues and integrate these into epigenetically stabilized gene expression patterns during lineage specification. The underlying mechanisms to ensure this necessary epigenetic plasticity are therefore active and highly dynamic in embryonic stem cells (ESC). One of those mechanisms includes the thymine DNA-glycosylase (TDG) and the downstream factors of DNA base excision repair (BER), which were shown to be involved in the dynamic, locus-specific regulation of DNA methylation. Following the activity of dioxygenases of the ten-eleven-translocation (TET) family that iteratively oxidize 5-methylcytosine (5-mC) to formyl- and carboxylcytosine (5-fC, 5-caC), TDG-induced BER provides a mechanism of active DNA demethylation and, thereby, impact gene regulation ( Cortázar *et al*., 2011; Cortellino *et al*., 2011; He *et al*., 2011; Ito *et al*., 2011; Schuermann *et al*., 2016; Shen *et al*., 2013; Weber *et al*., 2016). Studying the precise function of TDG (and BER) in epigenetic regulation during differentiation of stem cells, however, is challenging due to its multiple and interwoven interactions with other proteins like transcription factors and epigenetic modifiers ( Henry *et al*., 2016; Jacobs & Schär, 2012; Léger *et al*., 2014), the deregulation of which may cause a plethora of confounding effects. Also, genetic depletion of TDG itself is not compatible with embryonic development ( Cortázar *et al*., 2011; Cortellino *et al*., 2011). To reduce and circumvent such confounders in functional studies of TDG (and BER) in murine ESCs, we established and validated a *Tdg* minigene, introduced it into mice and derived a series of different ESC variants. The minigene carries, among other features, a *loxP*-flanked coding region of *Tdg*, allowing for a controlled and fast, 4-hydroxytamoxifen (OHT)-inducible depletion of TDG in the background of a homozygous disruption of the endogenous *Tdg*. By eliminating phenotypic divergence between TDG proficient and deficient ESCs, this versatile model facilitates precise functional and mechanistic investigations into TDG-mediated active DNA demethylation. This novel *Tdg* minigene introduced into mice as well as ESCs will facilitate future research in the field of active DNA demethylation *in vivo* and *in vitro.*

## Methods

### Ethics statement and Animal Work

All animal work was carried out in accordance with the Swiss Animal Welfare Act and the guidelines of the Swiss Federal Veterinary Office (SFVO) or with the UK Animals (Scientific Procedures) Act. Housing, breeding and experimentation of mice has been performed with the approval of the Cantonal Veterinary Office of Basel-Stadt (Licenses 10062-H, 1912) or was covered by a project license to Wolf Reik (80/1896) further regulated by the Babraham Institute Animal Welfare, Experimentation, and Ethics Committee. Mice were housed under specific pathogen free condition in individually ventilated cages under 12 h light/dark cycles at 22 ±2°C and 35–65% relative humidity. Animal and colony health was checked daily and quarterly, respectively. Sterilized diet (Kliba-Nafag Extrudate 3436) and water was provided *ad libitum*.

*Tdg ^tm1Psch^* mice ( http://www.informatics.jax.org/allele/MGI:5487834) were generated from E14 ESC (RRID:CVCL_C320) and backcrossed for more than 10 generations with female C57BL/6JRj mice, obtained at 7-8 weeks of age from Janvier Labs. A *Tdg* expression cassette (pTCO2-mTDGi.0, Addgene Plasmid #149429) was introduced as single copy into C57BL/6JRj mice by pronuclear injection and crossed into *Tdg ^tm1Psch^* mice. Genotyping was done by PCR with the HOT FIREPol (Solis BioDyne) on diluted crude DNA preparations (diluted by a factor of five with 10 mM Tris-HCl pH8) by boiling toe clips with 25 mM NaOH/0.2 mM EDTA for 1 h followed by neutralization with 40 mM Tris-HCl. Reactions were performed according to the manufacturer’s recommendation with 35 PCR cycles at 95°C for 30 s, 60°C for 30 s, and at 72°C for 60 s (for details about all used primers and annealing temperatures in PCR reactions, see *Extended data* Table S1) ( Schwarz, 2020).

### Generation of ESCs

ESCs were derived from male blastocysts from timed matings of male *miniTdg ^tg/tg^*// *Tdg* ^-/-^ mice with 2 super-ovulated female *Tdg* ^+/-^ mice and cultured on murine immortalized feeder cells in 2i medium with leukemia inhibitory factor LIF (Merck-Millipore) ( Ying *et al*., 2008).

To introduce the inducible Cre recombinase into the ROSA26 locus, 30 µg of the targeting vector (pROSA26-ERT2CreERT2, Addgene Plasmid #149436) was electroporated into 15×10 ^6^ mESCs using a gene pulser Xcell (Bio-Rad) at 240 V and 475 μF. Transgenic cells were selected with 8 and 5 µg/ml blasticidin for a week each, before colonies were picked and then amplified without selection pressure. From blasticidin-resistant colonies, genomic DNA was extracted with the “QIamp DNA mini” kit (Qiagen) and screened for a targeted integration at the 3’ junction of the ROSA26 locus by PCR with the Phusion polymerase (NEB) (see *Extended data,* Table S1) ( Schwarz, 2020), applying a general three-step cycling protocol: initial denaturation 95°C for 30 s, 35 times 95°C for 10 s, annealing for 20 s, 72°C for 15–80 s. PCR reactions were analyzed by gel electrophoresis followed by image acquisition with a U:Genius 3 system (Version 3.0.12.0, Syngene).

To evoke a disruption of the *Neo ^R^* reading frame by CRISPR/Cas9, two guide RNAs ((Neo ^R^)3: 5’-GCCGATCCCATATTGGCTGCAGG-3’, (Neo ^R^)4: 5’-GAAGGCGATGCGCTGCGAATCGG-3’, IDT) were designed and transfected with *Trans*IT-X2 (Mirus Bio) into the TDGiKO1 ESCs. RNP assembly was performed according to the manufacturers protocol (IDT). One day after transfection, single cells were sorted with a FACSaria IIIu (BD BioSciences) into 96-well plates coated with inactivated mouse fibroblasts, based on GFP expression from a co-transfected pEGFP-N1 plasmid (Clontech). Screening for the successful disruption of the *Neo ^R^* gene was done by PCR as described above and assaying the loss of the neomycin resistance with the Cell Counting Kit-8, according to the manufacturer’s instructions (CCK-8, Dojindo).

### Cell culture and differentiation

ESCs were cultured under a controlled atmosphere (37°C, 5% CO _2_, 95% humidity) in serum-free 2i medium with 1000 U/ml LIF, without antibiotics unless indicated otherwise. The 4-OHT (Sigma-Aldrich H7904) was dissolved in DMSO (stock 10 mM) and administered at indicated concentrations for 2 h.

Neuronal differentiation was performed as described before ( Bibel *et al*., 2007; Cortázar *et al*., 2011; Steinacher *et al*., 2019). Differentiation towards cardiomyocytes was performed by culturing ESCs in classical ESC medium (ESM) with 15% FCS without LIF in non-adhesive petri dishes (Greiner) for at least 10–14 days.

All pictures were taken with a DM IL LED Fluo microscope and MC170 HD camera (Leica) at 100x magnification.

### Molecular analysis of ROSA26 targeting and *miniTdg* excision

Probes for Southern blotting were generated by PCR amplification with Taq DNA Polymerase (Qiagen) from the ROSA26 targeting plasmid using the DIG DNA labeling mix (Roche). Southern blotting was done with 20 µg of genomic DNA, extracted by the “Genomic tip 100G” kit (Qiagen). Probes were hybridized according to the “DIG Application Manual for Filter Hybridization” by Roche. Signals were acquired by exposing the membrane to chemiluminescence detection films (Amersham/GE Healthcare) and digitalized by a CanoScan 8400F scanner with CanoScan Toolbox (Version 4.9.3).

Excision of the *miniTdg* gene by Cre was assessed by qPCR with the “Rotor-Gene SYBR Green PCR Kit” (Qiagen) on a Rotor Gene 3000 (Qiagen) system. After 95°C for 5 min, qPCR reactions followed a two-step cycling (40x) protocol: 95°C for 5 s, 60°C for 30 s, terminated by ramping from 60 to 95°C in 175 s to generate a melting curve. PCRs to exclude background recombination by Cre were done with NEB Phusion polymerase as described above.

### Gene expression analyses

Total RNA was extracted with the RNAeasy Mini Kit (Qiagen), including on-column DNase digest, and reverse transcribed with RevertAid First Strand cDNA synthesis kit (ThermoFisher) using oligo-dT primers. qPCR was performed as described above with marker-specific primers (see *Extended data*, Table S1) ( Schwarz, 2020).

Protein levels were analyzed by western blotting of 50 µg of NP-40 (or SDS) whole-cell extracts, separated by SDS-PAGE, onto a nitrocellulose membrane (Amersham) and immunodetection with anti-mTDG antibody (rabbit polyclonal L58, Lab P.Schär, dilution 1:10’000, 1 h at 33°C) and anti-GAPDH antibody (Sigma-Aldrich Cat# G9545, RRID:AB_796208, dilution 1:20’000, 1 h at RT). Chemiluminescence signals were detected with a Fusion FX7 system (Software version 16.15.0.0, Vilber).

### Detection of oxidized variants of 5-methylcytosine

DNA was extracted and purified with the Genomic tip 100G Kit (Qiagen). High-performance liquid chromatography–tandem mass spectrometry analysis was performed on 10 µg of genomic DNA as described before ( Weber *et al*., 2016).

### Statistical analysis

To test for statistical significance ( *p* ≤ 0.05), we performed two-tailed Student’s *t*-tests on the indicated number of replicates in Microsoft Excel (Version 16.0.4954.1000)

## Results and discussion

### Generation of a chimeric *Tdg* minigene and derivation of murine ESCs carrying a tamoxifen-inducible Cre recombinase

**Figure 1.**
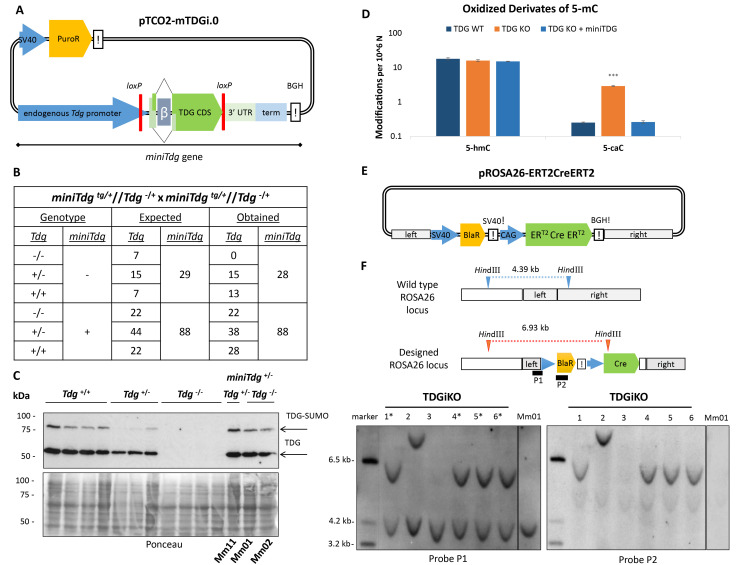
*miniTdg* complementation of *Tdg* ^-/- ^mice and mESCs. ( **A**) Synthetic *miniTdg* gene on the plasmid integrated to a random genomic locus by pronuclear injection. *miniTdg* consisting of 6 kb endogenous *Tdg* promoter, the *Tdg* coding sequence (CDS) including a chimeric splice donor ( *Tdg* exon 1 of transcript variant 2 NM_172552.4, rabbit β-globin intron), to allow for alternative splicing, 3 kb of the 3’ untranslated region (3’ UTR) and terminator (term) of *Tdg*, and the bovine growth hormone terminator sequence (BGH!). loxP sites are indicated in red. ( **B**) Expected and obtained genotype distribution in offspring (n=116) from crosses of heterozygous mice. ( **C**) Top: Immunoblot for TDG of whole-cell SDS-extracts from derived ESCs with different genotype. Bottom: Loading control by Ponceau staining of the membrane. ( **D**) Accumulation of oxidized 5-mC derivates measured by HPLC-MS/MS in ESCs with the indicated genotype. For each genotype, means and standard deviations of two independent clones with 1 or 2 repeated measurements are shown. Asterisks indicate *p*-values of Student’s t-test, compared to TDG WT: *** *p*-value < 0.001. ( **E**) Scheme of the targeting construct pROSA26-ERT2CreERT2 for the ROSA26 locus with the tamoxifen-inducible Cre recombinase ( Matsuda & Cepko, 2007). ERT2-Cre-ERT2 expression is under the control of the synthetic cytomegalo-virus/chicken-actin/beta-globin promoter (CAG). Homology arms for ROSA26 targeting are indicated in light grey. A blasticidin resistance cassette (BlaR) for positive selection is under the control of the SV40 promoter and terminator. ( **F**) Predicted restriction pattern of ROSA26 WT and integration events (top) and fragments detected by Southern blotting (bottom). Left: The hybridization probe (P1) locating to the left ROSA26 homology arm detected a genomic fragment of 6.9 kb. The 4.4 kb fragment represents the wild-type ROSA26 allele. Right: Detection of possible off-target events using a second probe (P2) locating to the blasticidin selection marker within the targeting construct. See *Underlying data* for the raw data behind this figure ( Schwarz, 2020).

To facilitate genetic manipulation of the *Tdg* gene (~28 kb including the putative promoter), we first constructed a synthetic *Tdg* minigene ( *miniTdg,* ~11 kb) ( Figure 1A). The minigene consists of the endogenous *Tdg* promoter and terminator sequences flanking the *Tdg* coding sequence (CDS). It also includes the rabbit β-globin intron at the authentic position of the first intron of the *Tdg* transcript variant 2 (Genebank NM_172552.4). This intron allows for the expression of the two naturally occurring *Tdg* splice variants ( Um *et al*., 1998). In addition, the *miniTdg* contains two *loxP* sites in the same orientation for Cre-recombinase-mediated excision of the coding region. A plasmid carrying the *miniTdg* gene (pTCO2-mTDGi.0) was introduced into C57BL/6 mice at a random genomic location by pronuclear injection and offspring carrying the minigene were crossed into heterozygous *Tdg* ^tm1Psch^ mutant mice ( Kunz *et al*., 2009). When breeding heterozygous mice for the *miniTdg* transgene and the *Tdg* KO allele, the genotypes of offspring followed the expected Mendelian pattern for two loci ( Figure 1B). The *miniTdg* allele segregated with a 3:1 ratio, consistent with a random integration into a single genomic locus. About 25% of born mice with the *miniTdg* gene were homozygous for the *Tdg* ^tm1Psch^ KO allele while no homozygous *Tdg* KO mouse was obtained without the transgene. This shows that the *miniTdg* transgene is fully functional in complementing the developmental defects and lethality of homozygous *Tdg* ^tm1Psch^KO embryos ( Cortázar *et al*., 2011; Cortellino *et al*., 2011). *MiniTdg* complemented mice were healthy and fertile with normal lifespan.

Three ESC populations were then derived from male blastocysts from crosses of *miniTdg ^tg/tg^*// *Tdg* ^-/-^ with *Tdg* ^+/-^ mice ( *Extended data*, Table S2: Mm11, Mm01, Mm02) ( Schwarz, 2020). In these ESCs, TDG protein levels were similar to those in wild-type ESCs ( Figure 1C), indicating that the *miniTdg* gene is regulated as the endogenous *Tdg*. Furthermore, no silencing of the transgene was noticed over several generations of animal breeding or culturing of ESCs. To assess functional integrity at the molecular level, we measured the accumulation of oxidized 5-methylcytosine (5-mC) derivates, 5-hydroxymethylcytosine (5-hmC) and 5-carboxylcytosine (5-caC), the substrate for excision by TDG ( Maiti & Drohat, 2011; Shen *et al*., 2013) ( Figure 1D). Mass spectrometry showed that TDG deficient ESCs accumulated 5-caC as expected, while ESCs expressing TDG solely from the *miniTdg* gene showed 5-caC levels similar to those in wild-type ESCs.

To allow for a controlled excision of the *miniTdg* coding region, we introduced a tamoxifen-inducible Cre recombinase expression cassette ( Figure 1E) into the non-essential ROSA26 locus ( Soriano, 1999). Screening 60 blasticidin-resistant colonies by PCR for targeted integration at 3’ ROSA26 locus yielded six clones (TDGiKO1-6). Southern blotting confirmed correct heterozygous integration of the complete ER ^T2^-Cre-ER ^T2^ cassette ( Matsuda & Cepko, 2007) for four of the six clones ( Figure 1F, left, asterisks). Potential additional off-target integrations were tested and excluded by hybridizing a second probe within the transgene ( Figure 1F, right). Original Southern blotting images are available as *Underlying data* ( Schwarz, 2020).

Taken together, we established a *miniTdg* gene that complements expression and function of the endogenous *Tdg* gene *in vivo* and *in vitro.* The functional integrity of the transgene is confirmed by rescuing phenotypes of *Tdg* KO defects at the systemic and cellular level, namely the rescue of embryonic lethality and suppression of 5-caC accumulation. The introduction of a Cre-recombinase to the ROSA26 locus, without off-targeted events, will allow conditional inactivation of the *miniTdg* in ESCs.

In addition, we generated ESC clones that ease further genetic manipulations involving selectable marker genes. We eliminated the neomycin resistance gene, which was introduced at the endogenous *Tdg* locus to generate the original *Tdg* KO allele ( Kunz *et al.*, 2009), by applying a CRISPR-guided Cas9 nuclease. Using two guide RNAs targeting the 5’ and 3’ end of the *Neo ^R^* gene, we generated two ESC clones (TDGiKO1.1 and TDGiKO1.2) that bear a deletion of 776 and 767 bp, respectively, and are no longer resistant to neomycin ( *Extended data*, Figure S1A-C) ( Schwarz, 2020).

### Induction of the Cre recombinase leads to fast and efficient depletion of TDG in ESCs

**Figure 2.**
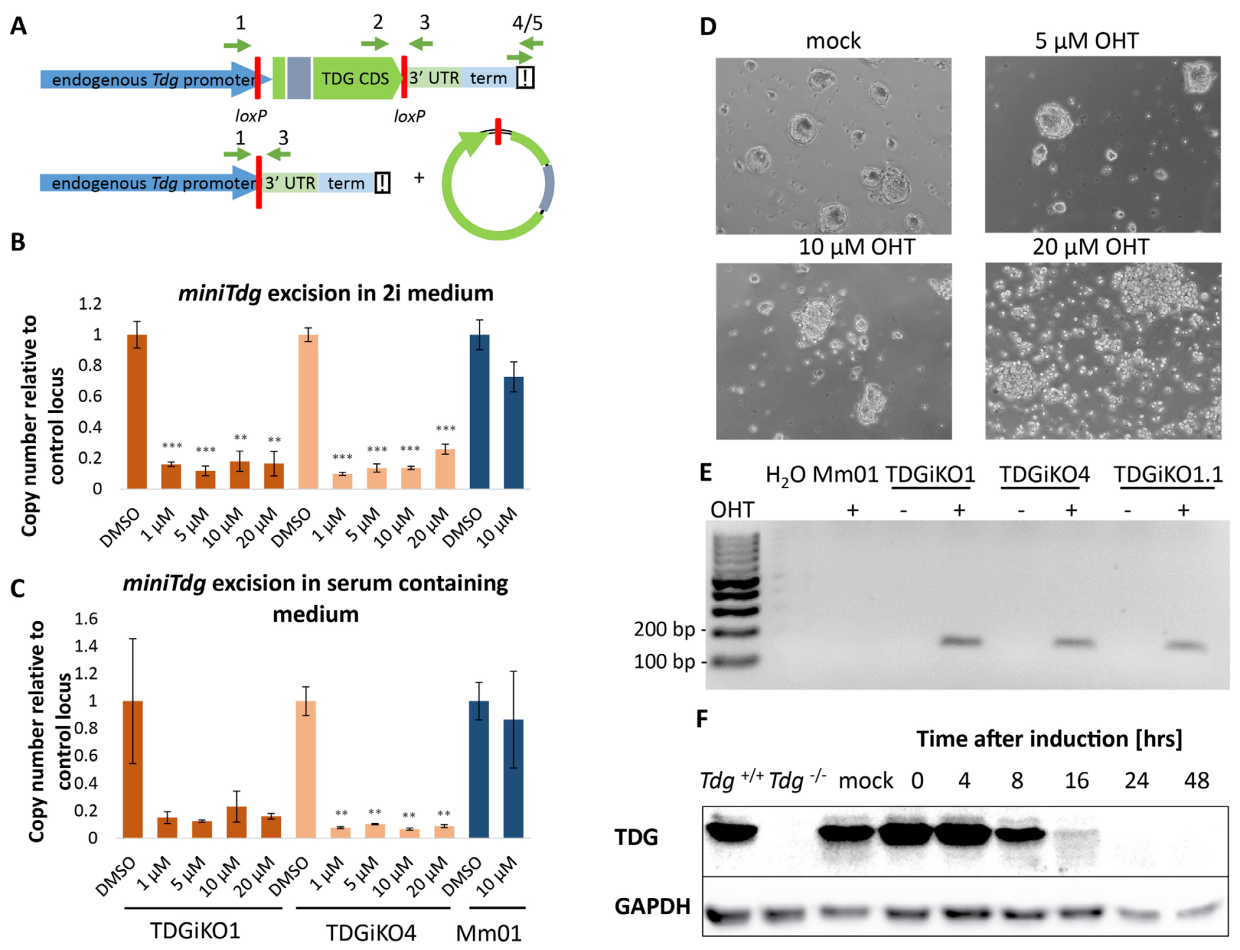
Cre induction for two hours results in the depletion of TDG within 24 hours without affecting cell viability. ( **A**) Scheme of the *miniTdg* cassette before (top) and after (bottom) Cre-mediated recombination. Position of PCR primers are indicated with green arrows. ( **B**) Quantitation of *miniTdg* excision upon OHT administration in TDGiKO1, TDGiKO4 and parental ESCs without the Cre recombinase (Mm01). Genomic DNA of cells was extracted two days after OHT treatment with indicated concentrations for 2 hours. Copy number of the *miniTdg* is measured by qPCR using primers 2 and 3, normalized to the nearby terminator region (primers 4/5) and a control locus on chromosome two. Shown are means and standard error from technical quadruplicates per clone. Asterisks indicate *p*-values of Student’s t-test, compared to DMSO control: ** *p*-value < 0.01, *** *p*-value < 0.001. ( **C**) Quantitation of *miniTdg* excision as in ( **B**), but Cre induction executed in ES medium containing 15% FCS. ( **D**) Phase-contrast images of TDGiKO1 ESCs in 2i medium, two days after OHT treatment for two hours at indicated concentrations. ( **E**) Detection of Cre recombination events (158 bp) by PCR using primers 1 and 3 ( **A**) before and after addition of OHT. ( **F**) Time-course assessment of TDG protein levels expressed from the *miniTdg* gene. Immunodetection with an anti-TDG antibody was performed with western blotted NP-40 cell extracts of 2i cultivated TDGiKO1 ES cells after Cre induction by 1 µM OHT for 2 h. See *Underlying data* for the raw data behind this figure ( Schwarz, 2020).

To make the inducible TDG depletion ( Figure 2A) applicable to differentiation experiments that often depend on complex and strict cell culture procedures, we aimed at establishing a short Cre-induction procedure minimizing any interference with standard differentiation conditions. Titrating 4-Hydroxytamoxifen (OHT) and measuring *miniTdg* excision by quantitative PCR, we identified OHT concentrations of 1-5 µM in serum-free 2i ( Figure 2B) and 5-10 µM OHT in serum-containing medium ( Figure 2C) to yield maximal excision efficiencies without impairing cell viability ( Figure 2D). We note that lower OHT doses (down to 100 nM) for longer duration (up to 48 h) may be applied as well, depending on the experimental procedure and objective. While the *miniTdg* excision product is readily detectable by PCR in different TDGiKO ESCs after OHT treatment, no unintentional background activity of the Cre recombinase is detectable before OHT induction ( Figure 2E). Following the kinetics of TDG depletion by SDS-PAGE/immuno-detection, we found that TDG was reduced below detection limit (<2% of endogenous TDG) 24 h after Cre induction for 2 h ( Figure 2F and *Extended data,* Figure S1D) ( Schwarz, 2020).

We also assessed the tissue specific induction of Cre-mediated *miniTdg* excision *in vivo*. We crossed *miniTdg* mice with mice expressing Cre-recombinase under the control of a *FoxN1-*promoter ( http://www.informatics.jax.org/allele/key/62500) which drives expression in thymic epithelial cells (TECs), but not in thymic cells (TCs). We observed that the mRNA and protein expressed from the *miniTdg* are reliably depleted in TECs expressing *FoxN1-*driven Cre, whereas TDG levels are much less affected in TCs ( *Extended data,* Figure S1E/F) ( Schwarz, 2020).

These data demonstrate the functionality and tight regulation of the inducible *miniTdg* KO model in murine ESCs as well as in mice. A short-time Cre induction with 1–5 μM or 5–10 μM OHT (in 2i+LIF or 15% FCS-containing medium, respectively) is sufficient to mediate excision of the *miniTdg*, resulting in depletion of TDG below detection within 24 hours. This rapid depletion of TDG is presumably facilitated by the cell cycle-regulated proteasomal degradation of TDG via the ubiquitination at its PCNA-interacting peptide (PIP) motif ( Hardeland *et al*., 2007; Shibata *et al*., 2014).

### Molecular and cellular phenotypes of TDG depletion in TDGiKO ESCs

**Figure 3.**
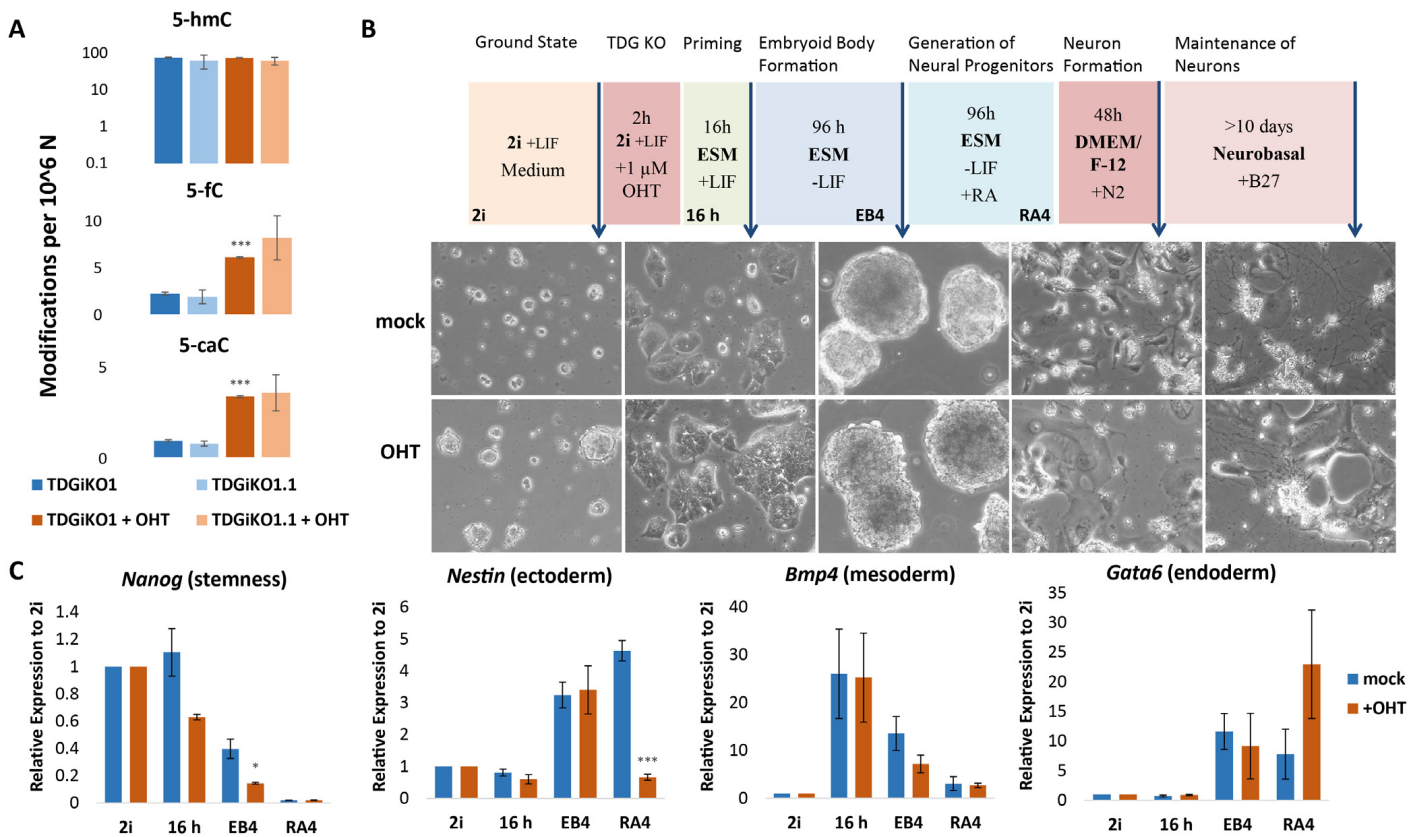
TDGiKO ESCs express a functional TDG and are proficient for cell lineage commitment. ( **A**) Measurement of oxidized derivates of 5-mC in selected ESCs by HPLC-MS/MS, cultivated in 2i medium + LIF, one week after TDG depletion by 1 µM of OHT for 2 h. Shown are the means and standard deviation of 2-3 biological replica per condition. ( **B**) Scheme of the neural differentiation protocol ( Bibel *et al*., 2007), adapted with a 16 h priming step in ESC medium for ESCs grown in 2i medium. Representative phase-contrast images of TDGiKO1 cells with (+OHT) and without (mock) TDG depletion at the differentiation stages indicated (arrows). ( **C**) Expression of pluripotency and germ layer marker genes were assessed by qRT-PCR in TDGiKO1 cells at the stages of differentiation as marked in ( **B**). Expression was normalized to the housekeeping genes *Rps13* and *Eef1a1*. Shown are means and standard errors of 3 biological replicates. Asterisks indicate *p*-values of Student’s t-test, compared to the non-induced condition: * *p*-value < 0.05, *** *p*-value < 0.001. See *Underlying data* for the raw data behind this figure ( Schwarz, 2020).

To validate the molecular phenotype of TDG depletion in our newly derived TDGiKO ESCs (TDGiKO1 ESCs and derived Neo ^R^-deleted TDGiKO1.1), we again assessed changes in levels of oxidized 5-mC derivates, 5-formylcytosine (5-fC) and 5-caC following Cre-mediated *miniTdg* deletion. One week after induction, we measured a 4.4 and 4.2-fold accumulation of the TDG substrates 5-fC and 5-caC in TDG-depleted TDGiKO ESCs, respectively, but no changes of 5-hydroxymethyl cytosine (5-hmC), which is not recognized by TDG ( Figure 3A). This accumulation reflects well the previously reported measurements in constitutive *Tdg* KO cells ( Shen *et al*., 2013; Steinacher *et al*., 2019).

Finally, we re-tested the pluripotency and differentiation capacity of some of the engineered TDGiKO ESC clones ( *Extended data*, Table S2) ( Schwarz, 2020), applying a protocol for all-trans retinoic acid (RA) induced *in vitro* differentiation towards the neuronal lineage ( Bibel *et al.*, 2007) with minor adaptations. TDG depletion was induced just before an additional culturing period of 16 h in serum containing medium, which promotes the transition from the ground (cultured in 2i medium) to the primed ESC state ( Figure 3B). Formation of embryoid bodies and neural progenitor cells was triggered by LIF withdrawal and later the addition of RA, before terminal differentiation was induced by replating the cells in media for neural cells. When subjecting TDG proficient TDGiKO1 ESCs to neural differentiation, we observed an upregulation of the neural marker *Nestin* ( Figure 3B, C) and, ultimately, the formation of neuron-like cells ( Figure 3B). As expected during embryoid body (EB) formation, LIF withdrawal resulted in an induction of lineage-specific genes of all three germ layers and the repression of the pluripotency marker gene *Nanog*. This indicates the ability of the TDG proficient TDGiKO ESCs to commit to cell types of all germ layers. In support of this, continued cultivation of non-induced TDGiKO1 and TDGiKO1.1 in ESM without LIF led to a spontaneous differentiation to cardiomyocytes (mesodermal cell type) forming beating organoids (see *Extended data* Videos 1 and 2) ( Schwarz, 2020). While the early initiation of lineage-specific genes seemed to be unaffected by TDG depletion prior to differentiation, the formation of neuron-like cells ( Figure 3B, bottom) was impaired. This deficiency to stably commit to and/or maintain the neuronal lineage was indicated by a low number of neuron-like cells formed and the loss of cells with *Nestin* expression following RA application. Upon loss of TDG, we furthermore observed a significant difference in the downregulation of the pluripotency marker *Nanog* as well as and dysregulation of the endodermal marker *Gata6* ( Figure 3C).

Thus, when subjecting the TDG-depleted TDGiKO ESCs to *in vitro* differentiation, we obtained data consistent with previous observations in constitutive *Tdg* KO ESCs ( Steinacher *et al*., 2019). This highlights the usability of the newly established ESCs in TDG and BER-related research, with the advantage of eliminating clonal divergences between TDG proficient and deficient cells. While here, we focused on the reproduction of published *Tdg* KO phenotypes, the fast and reliable depletion of TDG in the TDGiKO ESC model now provides a tool for in-depth execution point analysis of TDG-BER mediated active DNA demethylation during cell differentiation or in any other context of interest. Furthermore, the *loxP* sites flanking the entire coding region of the *miniTdg* gene enable recombinase-mediated cassette exchange to introduce TDG separation of function variants devoid of catalytic function or harbour mutations that ablate posttranslational modifications ( Hardeland *et al*., 2000). Such an exchange with functional TDG variants or fusion proteins is not easily applicable with other available murine conditional *Tdg* knockout systems (e.g.: Cortellino *et al.*, 2011; Hassan *et al.*, 2017; Hu *et al.*, 2014; Song *et al.*, 2013). Additionally, the availability of mice carrying the *miniTdg*, which can be combined with Cre-driver mice of interest by crossing, allows the translation of *in vitro* observations into *in vivo* settings. All in all, we provide a versatile, well-controlled and validated toolbox ( *Extended data*, Table S2) ( Schwarz, 2020) to facilitate functional and mechanistic research on TDG-mediated active DNA demethylation and BER in cell culture and animal models.

## Data availability

### Underlying data

Zenodo: Inducible TDG knockout models to study epigenetic regulation. http://doi.org/10.5281/zenodo.4075154 ( Schwarz, 2020).

This project contains the following underlying data:

- Raw_Data_Figure1.zip. Database excerpt for genotyping of mouse crossings, original pictures of Southern and western blots and MS-quantification of oxidized mC derivates (also for figure 3).
- Raw_Data_Figure2.zip. qPCR data of *miniTdg* excision, original pictures of OHT-treated ESCs and western blots.
- Raw_Data_Figure3.zip. Original pictures of differentiating cells and qPCR data of pluripotency and germ line markers.
- Raw_Data_Extended_Figure.zip. Sequencing data for *Neo ^R^* excision and predicted off-targets, and predicted off-targets, absorption values of G418-treated ESCs, qPCR data for *miniTdg* excision and original pictures of western blots.

### Extended data

Zenodo: Inducible TDG knockout models to study epigenetic regulation. http://doi.org/10.5281/zenodo.4075154 ( Schwarz, 2020).

This project contains the following extended data:

- Extended Figure and Tables.pdf. Supplementary information as referenced in the text.
- Extended_Video_1_TDGiKO1.mp4. Video of “beating bodies” from an ESC clone.
- Extended_Video_2_TDGiKO1.2.mp4. Video of “beating bodies” from a second ESC clone.

Data are available under the terms of the Creative Commons Attribution 4.0 International license (CC-BY 4.0).
