# Supplementary material for: Inducible TDG knockout models to study epigenetic regulation
Source: F1000Res. 2020 Oct 19;9:1112. Originally published 2020 Sep 9. [Version 2] doi: 10.12688/f1000research.25637.2 (PMC7527862; doi:10.12688/f1000research.25637.2)
Supplement: Peer review history

# Peer review: Reviewer response for version 2

**DOI:** 10.5256/f1000research.30222.r73317

**Published:** 2020-12-02

**Authors:** Sebastian Bultmann

**Affiliations:** 1 Department of Biology II and Center for Integrated Protein Science Munich (CIPSM), Ludwig-Maximilians-Universität München, Munich, Germany

The authors have addressed all concerns.

Is the rationale for developing the new method (or application) clearly explained?

Yes

Is the description of the method technically sound?

Yes

Are the conclusions about the method and its performance adequately supported by the findings presented in the article?

Yes

If any results are presented, are all the source data underlying the results available to ensure full reproducibility?

Yes

Are sufficient details provided to allow replication of the method development and its use by others?

Yes

Reviewer Expertise:

Epigenetics, embryonic stem cells, gene editing

I confirm that I have read this submission and believe that I have an appropriate level of expertise to confirm that it is of an acceptable scientific standard.

# Peer review: Reviewer response for version 1

**DOI:** 10.5256/f1000research.28295.r72019

**Published:** 2020-10-07

**Authors:** Sebastian Bultmann

**Affiliations:** 1 Department of Biology II and Center for Integrated Protein Science Munich (CIPSM), Ludwig-Maximilians-Universität München, Munich, Germany

In the manuscript entitled "Inducible TDG knockout models to study epigenetic regulation" the authors present new model system for the conditional depletion of TDG. The authors employ a strategy in which a loxP flanked mini gene is introduced into a TDG KO background. In combination with an inducible Cre introduced into the ROSA26 locus the authors convincingly show the rapid end efficient depletion of TDG. Finally, the authors validate their system by investigating the effect of acute TDG depletion on differentiation. 

 In summary, the study presents a clever strategy for induced deleption, is well thought through and scientifically sound. 

 I have only two minor points the authors should address:

1. Is the TDG mini gene randomly integrated into the genome? The authors should make this clear and if possible comment on any observation regarding silencing of the transgene during long-term culture.
2. The section "molecular and cellular phenotypes" is a little confusing. The authors should make clear in the text when they are talking about induced (TDG depeltion) or uninduced (control) cells. The authors should clearly explain the experimental setup shown in 3B in the text. In particular in the second paragraph of this section the authors start describing the control cells and then suddenly about TDG depleted cells. 

Is the rationale for developing the new method (or application) clearly explained?

Yes

Is the description of the method technically sound?

Yes

Are the conclusions about the method and its performance adequately supported by the findings presented in the article?

Yes

If any results are presented, are all the source data underlying the results available to ensure full reproducibility?

Yes

Are sufficient details provided to allow replication of the method development and its use by others?

Yes

Reviewer Expertise:

Epigenetics, embryonic stem cells, gene editing

I confirm that I have read this submission and believe that I have an appropriate level of expertise to confirm that it is of an acceptable scientific standard.

# Response

**Published:** 2020-10-12

**Authors:** David Schürmann

**Affiliations:** University of Basel, Switzerland

We thank Dr Bultmann for the evaluation of our manuscript. We appreciate his comments helping to improve it. Accordingly, the following adaptations were made :

1. The first point was also raised by Dr Torchia (reviewer 2) and we describe better the random integration of the *miniTdg* gene. We also added a note about the transcriptional stability of the transgene in chapter 1 of the Result section.
2. We apologize for the confusing description of our results. We now clearly indicate whether we refer to TDG proficient or deficient cells. We also added an outline of the experimental setup for the neural differentiation.

# Peer review: Reviewer response for version 1

**DOI:** 10.5256/f1000research.28295.r71089

**Published:** 2020-09-30

**Authors:** Joseph Torchia

**Affiliations:** 1 Department of Biochemistry, Western University, London, ON, Canada

Thymine DNA Glycosylase (TDG) is a BER protein that is essential for the removal of 5-formylcytosine (5-fC) and 5-carboxylcytosine (5-fC) from genomic DNA. TDG knockout is embryonic lethal at E11.5 due to genome wide epigenetic instability. To circumvent embryonic lethality from a deletion of *Tdg*, the authors synthesized a mini- *Tdg* allele consisting of an optimized TDG codon arrangement for expression of two naturally occurring TDG splice variants. The authors introduced the mini- *Tdg* allele in a *Tdg* ^-/-^ background to generate *miniTdg ^tg/tg^/Tdg ^-/- ^*mice, which phenocopy and express TDG similarly to wild-type mice.  Furthermore, the TDG codons are flanked by *loxp* sites to allow for efficient spatial and temporal excision of *Tdg *in response to tamoxifen. The deletion of TDG in ESC’s results in a global upregulation in 5-caC, and *Tdg* null ESC’s fail to differentiate in response to retinoic acid both of which are consistent with previous literature.  Overall, the paper describes a novel mouse model to study the role of TDG dependent active DNA demethylation *in vitro *and *in vivo*.

 The paper is well written, and the data seems to be convincing. In addition, the experiments appear to be performed to a high standard. However, I feel addressing the following concerns would significantly strengthen the manuscript and increase the novelty of the mouse model:

**Major Comments:**

1. The authors need to site appropriate literature and/or provide data to address the claim that epigenetic instability resulting from a loss of TDG leads to complications in long-term ESCs cultures. Multiple studies, such as Hu *et al.* 2014 ^1^ (PMID 24529596) and Shen *et al.* 2013 ^2^ (PMID 23602152), have rigorously used *Tdg ^-/-^* mouse ESC’s without any mention of such complications. I feel the argument that the use of such a complicated system to study TDG deletion in MEFs and ESCs needs further clarification.
2. There are several published conditional knockout mouse models of TDGthat incorporate *loxp *sites in the endogenous *Tdg *allele. These mouse models, and their potential drawbacks, need to be discussed to provide a stronger argument for the use of mini- *Tdg *allele approach employed in this manuscript.
3. Its unclear why it was necessary to remove the neo cassette to generate the TDGiKO1.1 and TDGiKO1.2 ESC clones? The use of CRISPR can introduce complications such as off-target mutations and clonal selection that can introduce variability in the model.

**Minor comments and questions:**

1. Figure 1D, 2B, 2C, 3A are all lacking statistical analysis. Please use the appropriate nomenclature to indicate statistical significance.
2. Please describe the sample size for LC-MS/MS experiment outlined in figure 1D and Figure 3A.
3. Figure 2F: Why is TDG blot not showing a doublet. The SUMOylated TDG band is missing.
4. Why was 4-OHT employed at such a high concentration? In my opinion 100 nM to 500 nM should be more than sufficient to induce excision.
5. Was the mini- *Tdg *allele integrated in the mouse genome? If so, where did the integration occur?

Is the rationale for developing the new method (or application) clearly explained?

Partly

Is the description of the method technically sound?

Yes

Are the conclusions about the method and its performance adequately supported by the findings presented in the article?

Yes

If any results are presented, are all the source data underlying the results available to ensure full reproducibility?

Partly

Are sufficient details provided to allow replication of the method development and its use by others?

Yes

Reviewer Expertise:

Epigenetics, Biochemist

I confirm that I have read this submission and believe that I have an appropriate level of expertise to confirm that it is of an acceptable scientific standard.

## References

[1] : Tet and TDG mediate DNA demethylation essential for mesenchymal-to-epithelial transition in somatic cell reprogramming. *Cell Stem Cell*.2014;14(4) : 10.1016/j.stem.2014.01.001 512-22 10.1016/j.stem.2014.01.001 24529596

[2] : Genome-wide analysis reveals TET- and TDG-dependent 5-methylcytosine oxidation dynamics. *Cell*.2013;153(3) : 10.1016/j.cell.2013.04.002 692-706 10.1016/j.cell.2013.04.002 23602152PMC3687516

# Response

**Published:** 2020-10-12

**Authors:** David Schürmann

**Affiliations:** University of Basel, Switzerland

We thank Dr Torchia for the critical reading of our manuscript and his valuable suggestions for improvement, which helped a lot to strengthen our conclusions. We revised the manuscript according to his major and minor comments.

 Major comments:

1. We understand the reviewers comment that this statement is rather speculative and currently poorly supported by data. We therefore removed it in the revised version. On the other hand, we observed in our experiments with constitutive TDG KO ESCs subtle and rather global differences (e.g. cell division time), which we do not yet fully understand. They could originate from clonal adaptation but also from TDG-driven epigenetic mechanisms. However, to be able to discriminate between these possibilities was the reason for the development of the here presented inducible TDG depletion system. Apart from the not yet ruled out role of TDG as repair enzymes for damaged DNA, the increase of fC and caC might affect DNA-protein interactions and consequently chromatin landscaping. Irrespective of these hypothetical scenarios, where long-term culturing of cells could result in both a genetic as well as an epigenetic drift, we feel that an isogenic system is always the preferable experimental system, even when it seems to be more complicated and laborious.
2. We are aware that several other conditional *Tdg* allele were generated and available as well, which are genetically easier to handle and could perfectly serve their experimental purposes. In fact, before having started to engineer our complementing *miniTdg* system, we intended but failed to introduce loxP sites at the extremities of the endogenous *Tdg* coding region to be able to delete the entire gene, omitting putative dominant truncated transcripts. We suspect that besides a poor accessibility of some regions of the *Tdg* gene, the multitude of pseudogenes consisting basically of *Tdg* cDNA sequences interferes with successful genome engineering in certain circumstances as they may act as off-targets, not only for classical gene targeting but also CRISPR/Cas9-based approaches. Having a functional *miniTdg* gene, in which the entire coding region is flanked by loxP sites, not only allows for an inducible TDG depletion but also enables further engineering steps in a flexible manner by Cre-mediated cassette exchange (although not designed specifically for this purpose). We refer to other available conditional *Tdg* allele in the revised version of our manuscript. In the last paragraph, furthermore, we point to this advantage and possibility. This was, by the way, one of the reasons why we deleted the neomycin resistance gene (see response to comment 3 below), allowing for the use of this selectable marker for such a replacement strategy.
3. We utterly agree with the reviewer about the potential drawbacks of the CRISPR/Cas9 genome editing approach. Therefore, we added our off-target analysis for the two ESC clones generated in the “Extended data”.
  In fact, most of the here presented data from cultured cells and *in vivo* are obtained from the systems without the additional deletion of the neomycin resistance gene (except LC-MS/MS data in Fig. 3A). For the sake of completeness of our system, we included these two ESC clones as they might be of interest when additional selection marker-based transgene integration is intended. These notions may have been presented ambiguously in the first version and are now improved by slightly rephrasing and restructuring of the respective paragraphs.
  In any case, the possibility to work with the inducible TDG depletion systems ensures an isogenic background, at least for the role of TDG, which could be validated in different in ESC clones. Although one can never completely rule out some issues of clonal selection, we still consider it an advantageous approach.

Minor comments and questions:

1. We appreciate reviewer’s pointing to the missing statistical indicators, which we now added.
2. More detailed information about the analysis by LC-MS/MS was added into the figure legends and the respective paragraph in the Methods section.
3. This is a very attentive comment by the reviewer. In contrast to Figure 1, indeed, the SUMO-modified TDG is not apparent in Figure 2F because we used NP-40 soluble protein extraction methods without the addition of specific inhibitors of SUMO proteases (f.e. N-Ethylmaleimide). As de-SUMOylation happens quite fast and efficiently during mild lysis condition even at 4°C, SUMOylated TDG is in our hands only detectable when immediate lysis in Laemmli buffer and/or detergent-based lysis buffers supplement by the inhibitor. We now added the technical information about the extraction method also in the figure legend.
4. We agree with the reviewer that lower 4-OH tamoxifen concentration might work as well and added a comment about suitable Cre induction conditions in the 2 ^nd^ chapter of the results.
  Based on an initial literature search, we found that commonly used concentrations of OHT are in the range of 100 nm – 10 µM. To get a maximal flexibility and timely controlled TDG depletion in experimental protocols of differentiation, we aimed at rather short durations of induction. Such single induction 4-OHT treatments of as short as 2 h are rarely found in literature and associated with limited recombination efficiency (e.g. Buelow et al. 2008), while lower concentrations are more frequently used for Cre induction for up to 48 h, sometimes also with repeated administration. For our purpose, we therefore opted for 4-OHT concentrations in the intermediate to high range but avoiding cytotoxic effects.
5. This is indeed a relevant point for a potential user of our system. The *miniTdg* gene was introduced by pronuclear injection of a plasmid that contained no homology arms for a targeted integration into the mouse genome. Thus, it is a random integration event as stated in the legend of Figure 1. We added now this information also the first result section.
  Unfortunately, our attempts to identify the genomic locus of integration by conventional molecular methods were so far not successful. Yet, we might get some hints from ongoing and future global NGS analyses, which would be amended to this paper as well as to the MGI entry.

# Peer review: Reviewer response for version 1

**DOI:** 10.5256/f1000research.28295.r71086

**Published:** 2020-09-21

**Authors:** Jonathan Sczepanski

**Affiliations:** 1 Department of Chemistry, Texas A&M University, College Station, TX, USA

In the method article “Inducible TDG knockout models to study epigenetic regulation” Schwarz *et. al* establish mice and mESC cell line that allows for inducible KO of TDG. Building on their prior work, the key features of this system is a TDG mini-gene that resides between two *loxP*-Cre recombinase sequences, along with a tamoxifen-inducible Cre recombinase expression cassette. The authors demonstrate this design achieves near complete (~98%) depletion of TDG upon tamoxifen-induction within 24 h. Importantly, the TDG mini-gene recapitulates the epigenetic and repair functions of endogenous TDG prior to depletion, and cell morphology and pluripotency are not altered until after tamoxifen-induced depletion. These molecular and cellular phenotype studies are particularly well done, and clearly demonstrate agreement between inducible TDG KO ESCs and previously reported constitutive TDG KO ESCs. Overall, this work is very rigorous, and the controls are carefully designed and extensive. I anticipate that inducible TDG KO will be a valuable tool for mechanistic studies aimed at uncovering the role of TDG in DNA epigenetics and repair. I recommend this paper for indexing without further revisions.

 Minor comment: ESC is written "ECS" in Figure 3 title.

Is the rationale for developing the new method (or application) clearly explained?

Yes

Is the description of the method technically sound?

Yes

Are the conclusions about the method and its performance adequately supported by the findings presented in the article?

Yes

If any results are presented, are all the source data underlying the results available to ensure full reproducibility?

Yes

Are sufficient details provided to allow replication of the method development and its use by others?

Yes

Reviewer Expertise:

DNA repair, base excision repair, DNA demethylation, thymine DNA glycosylase

I confirm that I have read this submission and believe that I have an appropriate level of expertise to confirm that it is of an acceptable scientific standard.

# Response

**Published:** 2020-10-12

**Authors:** David Schürmann

**Affiliations:** University of Basel, Switzerland

We are very grateful to Dr Sczepanski for taking the time to evaluate our manuscript and were pleased to learn about his overall approval and appreciation of our experimental work and data.

 The spelling mistake in the title of figure 3 was corrected.
